# Generation and Characterization of Recombinant Pseudorabies Virus Delivering African Swine Fever Virus *CD2v* and *p54*

**DOI:** 10.3390/ijms25010335

**Published:** 2023-12-26

**Authors:** Jianhui Wei, Chuancheng Liu, Xinyan He, Bilal Abbas, Qi Chen, Zhaolong Li, Zhihua Feng

**Affiliations:** 1 Fujian Key Laboratory of Innate Immune Biology, Biomedical Research Center of South China, College of Life Science, Fujian Normal University Qishan Campus, Fuzhou 350117, China; qbs20170107@yjs.fjnu.edu.cn (J.W.); qsx20211374@student.fjnu.edu.cn (C.L.); qsx20221410@student.fjnu.edu.cn (X.H.); bilalabbas857@gmail.com (B.A.); chenqi@fjnu.edu.cn (Q.C.); 2 Institute of Animal Husbandry and Veterinary Medicine, Fujian Academy of Agricultural Sciences, Fuzhou 350117, China

**Keywords:** African swine fever virus, pseudorabies virus, *CD2v*, *p54*, vaccine

## Abstract

African swine fever (ASF) leads to high mortality in domestic pigs and wild boar, and it is caused by the African swine fever virus (ASFV). Currently, no commercially available vaccine exists for its prevention in China. In this study, we engineered a pseudorabies recombinant virus (PRV) expressing ASFV CD2v and p54 proteins (PRV-∆TK-(CD2v)-∆gE-(p54)) using CRISPR/Cas9 and homologous recombination technology. PRV-∆TK-(CD2v)-∆gE-(p54) effectively delivers *CD2v* and *p54*, and it exhibits reduced virulence. Immunization with PRV-∆TK-(CD2v)-∆gE-(p54) neither induces pruritus nor causes systemic infection and inflammation. Furthermore, a double knockout of the *TK* and *gE* genes eliminates the depletion of T, B, and monocytes/macrophages in the blood caused by wild-type viral infection, decreases the proliferation of granulocytes to eliminate T-cell immunosuppression from granulocytes, and enhances the ability of the immune system against PRV infection. An overexpression of CD2v and p54 proteins does not alter the characteristics of PRV-∆TK/∆gE. Moreover, PRV-∆TK-(CD2v)-∆gE-(p54) successfully induces antibody production via intramuscular (IM) vaccination and confers effective protection for vaccinated mice upon challenge. Thus, PRV-∆TK-(CD2v)-∆gE-(p54) demonstrates good immunogenicity and safety, providing highly effective protection against PRV and ASFV. It potentially represents a suitable candidate for the development of a bivalent vaccine against both PRV and ASFV infections.

## 1. Introduction

African swine fever (ASF) is a highly contagious viral disease that leads to significant economic losses in the global swine industry. The severity of ASF clinical signs and the fatality rates varies depending on the animal species and the specific virus strains involved. Acute ASFV infection is associated with nearly 100% mortality [1,2]. ASFV, classified as a DNA virus, belongs to the *Asfivirus* genus within the Asfarviridae family. It is characterized by a 200 nm diameter icosahedral DNA structure comprising an envelope, capsid, inner capsule membrane, core shell, and inner core [3]. The viral genome is a linear double-stranded DNA molecule ranging from 170 kb to 190 kb in size, depending on the virus strain. It encodes between 150 and 200 viral proteins, encompassing 68 structural proteins and over 100 non-structural proteins [4,5].

Vaccination represents an effective strategy for preventing viral infection. Current vaccine options include live-attenuated, inactivated, recombinant virus, protein subunit, RNA, DNA, and virus-like particle (VLP) vaccines [6]. Live-attenuated or subunit vaccines have been developed in recent decades, and they show good protective effects. Live-attenuated vaccines have played an important role in epidemic control during and since the COVID-19 pandemic [7,8,9]. Despite this, concerns persist regarding their side effects and potential safety issues, including lymphadenopathy, recurrent fever, chronic viremia, persistent chronic infections, and the potential of virulence reversion [10,11,12,13]. In contrast, recombinant virus vaccines offer greater response predictability and higher efficacy [14]. Among them, the pseudorabies virus (PRV) is a good vector for developing multivalent vaccines [15].

Multivalent vaccines utilize PRV, a member of the Herpesviridae family, subfamily Alphaherpesvirinae, and genus Varicellovirus. As a vector that expresses major antigens of various swine pathogens, PRV has proven to be effective, as the expression of foreign genes does not impede viral replication [15]. Pseudorabies (PR), also known as Aujeszky’s disease, is caused by PRV. PRV has the capacity to infect numerous mammals, including mice, pigs, sheep, minks, and dogs, but the Suidae family serves as the only natural reservoir for the virus [16,17,18]. The disease was successfully controlled and eradicated from swine populations in most parts of China before 2011 through the inoculation of PRV-infected swine herds with the Bartha-K61 strain. However, since October 2011, outbreaks of PR among Bartha-K61-immunized swine populations have rapidly spread throughout China, resulting in significant economic losses to the swine industry [19,20]. Porcine subjects infected with the PRV variant exhibited more severe respiratory symptoms, neurological signs, organ damage, and a broader distribution of the virus, with higher viral loads in various organs compared to classical PRV cases [21]. Genomics analysis revealed that the re-emerging PRV belongs to a variant strain of genotype-2, and the Bartha-K61 vaccine fails to provide complete protection against these emerging PRV variants. Relative to the PRV classical strain, the PRV variants exhibit heightened virulence and infectiousness, faster transmission rates, more severe clinical symptoms, and increased mortality rates [20].

This underscores the need for new vaccines to avert the emergence of new epidemics. Several new vaccine strains, such as PRV TK^−^/gE^−^/gI^−^ (Fa), PRV-GD2013-ΔgI^−^/gE^−^, and PRV rHN1201TK^−^/gE^−^/gI^−^/11k^−^/28k^−^, have all demonstrated robust protective capabilities [22,23,24]. Among these, PRV TK^−^/gE^−^/gI^−^ (Fa) has been commercially employed for pseudorabies prevention.

PRV encodes over 70 open reading frames (ORFs), producing 70–100 proteins; however, only about 50 proteins are present in mature virus particles. Many of these genes are unnecessary for PRV replication, including *TK*, *gE*, and *gI*, which can be replaced with foreign genes [25]. Consequently, PRV is frequently used as a viral vector for the development of recombinant vaccines.

CD2v (*EP402R*) is the sole known viral homologue of CD2, sharing structural and functional similarities with the T lymphocyte surface adhesion receptor CD2. CD2v is a hemagglutinin protein encoded by ASFV that was previously linked to protective immunity [26]. The phenomenon of blood adsorption observed during ASFV infection depends on the expression of CD2v, which is likely to be caused by its extracellular domain [27]. ASFV infection in vitro inhibits the proliferation of peripheral blood lymphocytes, an effect that can be reversed by the deletion of the *CD2v* gene. Furthermore, *CD2v* deletion substantially hinders ASFV proliferation in lymphoid tissue and bone marrow, leading to a 100–1000 times reduction, thereby ameliorating viremia [28]. Thus, CD2v likely plays a role in immune evasion, tissue phagocytosis of ASFV, and immunosuppression. Blocking CD2v may reverse the host’s immunosuppressive state, enhancing its antiviral capacity [29]. CD2v has been employed in the development of subunit, DNA, and virus vector vaccines, which offer partial protection [30,31,32].

P54, encoded by *E183L*, serves as an ASFV inner-membrane protein. Its specific interaction with the 8 kDa light-chain cytoplasmic dynein (DLC8) is pivotal in virus internalization, transport, and assembly [33]. Moreover, p54 is involved in the binding of viral particles to target cells [34]. Antibody-induced production against p54 weakens the ability of ASFV to infect cells [34], highlighting its significance in subunit vaccine, nucleic acid, and viral vector vaccine research.

In this study, we engineered a recombinant PRV, PRV-∆TK-(CD2v)-∆gE-(p54), expressing both ASFV CD2v and p54 proteins using CRISPR/Cas9 technology, and assessed its safety, ability to stimulate humoral immune responses, and efficacy in providing protection in mice. These findings offer valuable insights for future vaccine development targeting both ASFV and PRV.

## 2. Results

### 2.1. Construction and Verification of Recombinant Pseudorabies Virus PRV-∆TK-(CD2v)-∆gE-(p54)

In order to develop a safe and effective vaccine against AFSV, PRV was used as the platform for vaccine construction. The recombinant pseudorabies virus PRV-∆TK-(CD2v)-∆gE-(p54), expressing both ASFV CD2v and p54 proteins, was constructed via homologous recombination using CRISPR/Cas9 technology.

**Figure 1:**
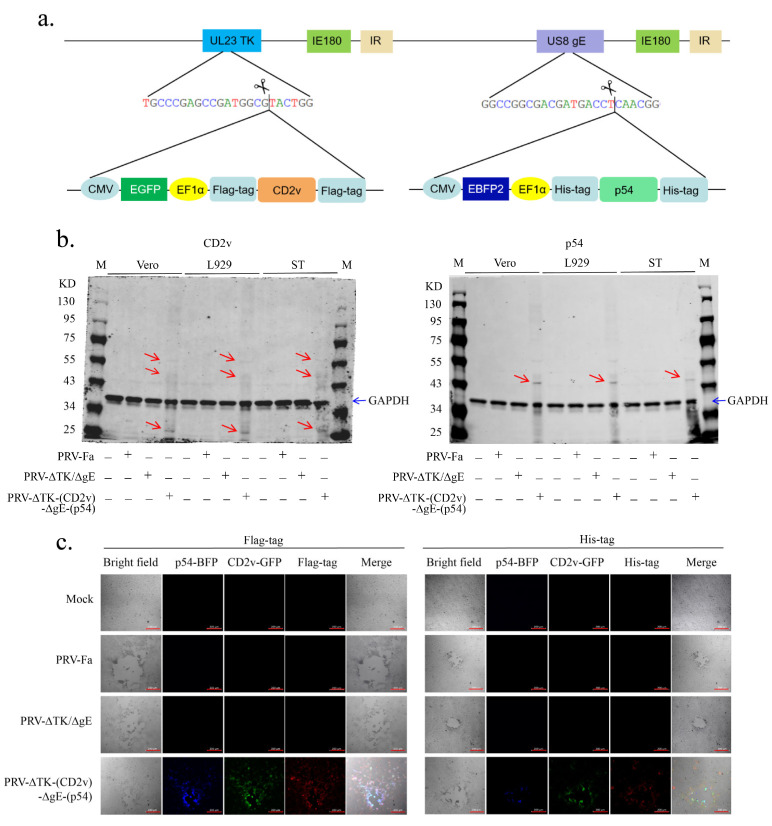
Construction and verification of recombinant virus PRV-∆TK-(CD2v)-∆gE-(p54). (**a**) Construct of recombinant virus PRV-∆TK-(CD2v)-∆gE-(p54); the scissors logo indicates the Cas9 protein cleavage site as well as the location of homologous template integration into the genome. (**b**) The expressions of the CD2v (**left panel**) and p54 (**right panel**) proteins in different cell lines after infection by the PRV-∆TK-(CD2v)-∆gE-(p54) recombinant virus were determined using Western blotting; 48 h post-infection, the cells were harvested and lysed, resolved through SDS-PAGE, and transferred to PVDF membranes; the membrane was blotted with anti-Flag-tag (CD2v) and anti-His-tag (p54) antibodies, respectively; the red arrow indicates the band of the target proteins; the blue arrow indicates the band of GAPDH. M, marker. (**c**) The CD2v (**left panel**) and p54 (**right panel**) protein expressions in Vero cells were determined under a fluorescence microscope at 24 h post-infection. Scale bar, 200 µm.

The configuration of this recombinant virus is illustrated in Figure 1a. The *EGFP* and *EBFP* were driven by the CMV promoter, and the *CD2v* and *p54* were driven by the EF1α promoter. For protein purification and subsequent verification, Flag-tag was added to both the N- and C-termini of CD2v, and 6xHis-tag was added to both the N- and C-termini of p54. To assess the ability of the recombinant virus to efficiently deliver and mediate transgene expression, we infected Vero cells with the wild-type virus PRV-Fa and the recombinant virus PRV-∆TK-(CD2v)-∆gE-(p54). Western blotting (Figure 1b) demonstrated the successful expressions of both CD2v (left) and p54 (right) in Vero, L929, and ST cells. Consistent with the Western blot results, we also confirmed the successful expression of CD2v and p54 in Vero cells following PRV-∆TK-(CD2v)-∆gE-(p54) infection using immunostaining (Figure 1c). As expected, Vero cells infected with PRV-Fa or PRV-ΔTK/ΔgE did not exhibit the expression of CD2v and p54 (Figure 1c).

In order to determine the genetic stability of PRV-∆TK-(CD2v)-∆gE-(p54), the virus was passaged for at least 40 generations, and the *CD2v* and *p54* genes were detected using PCR and Sanger sequencing. The results showed that the recombinant virus could stably inherit the *CD2v* and *p54* genes (Figure S1).

These findings demonstrate the successful construction of the recombinant virus PRV-∆TK-(CD2v)-∆gE-(p54) and its capacity to efficiently deliver and express CD2v and p54 proteins.

### 2.2. The Recombinant Pseudorabies Virus Has Reduced Virulence and Is Safe for Mice

**Figure 2:**
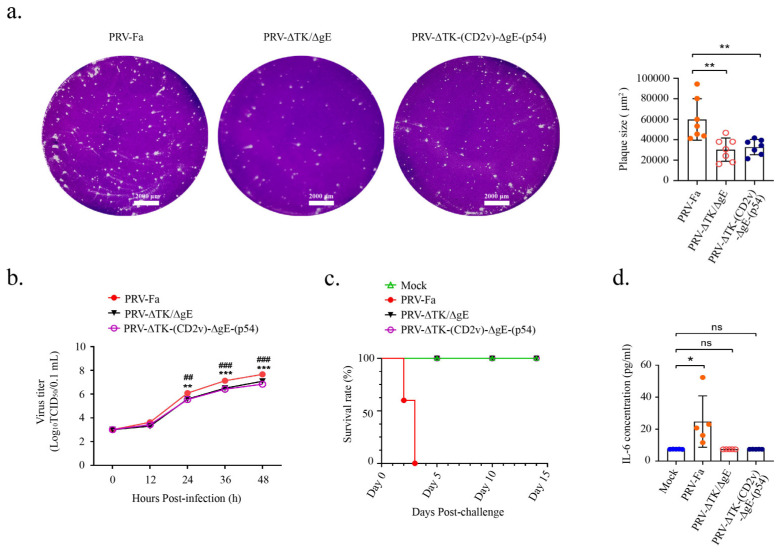
The PRV-∆TK-(CD2v)-∆gE-(p54) recombinant virus vaccine was characterized in vitro and in vivo. (**a**) Vero cells infected with PRV-Fa, PRV-∆TK/∆gE, or PRV-∆TK-(CD2v)-∆gE-(p54) were stained with crystal violet 36 h after infection. Seven plaques were randomly selected to measure their areas under the microscope (scale bar, 2000 µm). The size of the plaques was determined by ZEN blue 2.3 software. Left: a representative image of crystal violet staining; right: statistics of viral plaques. (**b**) A one-step growth curve was calculated and plotted using Karber’s method after the infection of Vero cells with PRV-Fa, PRV-∆TK/∆gE, or PRV-∆TK-(CD2v)-∆gE-(p54); virus titers in the supernatant were measured at 12 h, 24 h, 36 h, and 48 h. (**c**) Mice were infected with the wild-type virus (PRV-Fa), PRV-∆TK-∆gE, and PRV-∆TK-(CD2v)-∆gE-(p54), respectively, by intramuscular injection into the leg (100 μL, 5 × 10^5^ TCID_50_); PBS was used as a control. The survival rate of the mice in each group (*n* = 5) after infection with the corresponding virus was recorded for a total of 14 days; the survival rate in the group of mice infected with PRV-Fa was significantly decreased, whereas mice infected with PRV-∆TK/∆gE or PRV-∆TK-(CD2v)-∆gE-(p54) were comparable to negative control mice. (**d**) Interleukin (IL)-6 was measured with enzyme-linked immunosorbent assays; serum was collected at day 3 before the mice died in the group with PRV-Fa infection. There was a marked decrease in IL-6 secretion in PRV-∆TK/∆gE and PRV-∆TK-(CD2v)-∆gE-(p54) vs. PRV-Fa control. The data shown were obtained from 5 mice in each group. Error bars show ± SD. Statistical analysis was performed with the 2-tailed unpaired Student *t*-test. ## *p* < 0.01; ### *p* < 0.001 (# represent PRV-Fa vs. PRV-∆TK/∆gE), * *p* < 0.05; ** *p* < 0.01; *** *p* < 0.001 (* represent PRV-Fa vs. PRV-∆TK-(CD2v)-∆gE-(p54)), ns, not significant.

To assess the virulence of the recombinant PRV-∆TK-(CD2v)-∆gE-(p54) virus, one-step growth curves were determined in Vero cells for PRV-Fa, PRV-ΔTK/ΔgE, and PRV-∆TK-(CD2v)-∆gE-(p54). The strategy for infection is shown in Supplementary Figure S2. Following infection, crystal violet staining was applied, and viral plaques were quantified on the third day. The findings demonstrate a significant reduction in viral plaques produced by the recombinant virus (Figure 2a), indicating a weakened cell infection capacity. Moreover, utilizing Karber’s method, one-step growth curves were computed and graphed subsequent to Vero cell infection with the corresponding virus. Virus titers were assessed at 12 h, 24 h, 36 h, and 48 h, revealing a substantial reduction in titer for the recombinant virus (Figure 2b). These outcomes strongly suggest that double knockout of *gE* and *TK* leads to a diminished proliferation of PRV, thereby affirming the heightened safety profile of the PRV-∆TK-(CD2v)-∆gE-(p54) recombinant virus.

To assess the safety of the recombinant PRV-∆TK-(CD2v)-∆gE-(p54) virus, three groups of 6-week-old male C57BL/6 mice were infected intramuscularly (IM) with 5 × 10^5^ TCID_50_ PRV-Fa, PRV-ΔTK/ΔgE, or PRV-∆TK-(CD2v)-∆gE-(p54). The control group received a PBS vaccination. On the third day post-infection, severe pruritus was observed in the PRV-Fa group but not in the PRV-ΔTK/ΔgE- or PRV-∆TK-(CD2v)-∆gE-(p54)-infected groups, indicating that the insertion of *CD2v* and *p54* did not alter the pathogenicity of PRV-ΔTK/ΔgE.

Furthermore, the survival rate of mice in each group was monitored for a total of 14 days post-infection. The survival rate in the PRV-Fa-infected group was significantly decreased, while the mice infected with PRV-ΔTK/ΔgE or PRV-∆TK-(CD2v)-∆gE-(p54) exhibited a similar rate to the negative control group (Figure 2c). In the PRV-Fa-infected group, all mice died on the third day of inoculation, whereas mice infected with either PRV-ΔTK/ΔgE or PRV-∆TK-(CD2v)-∆gE-(p54) survived until they were sacrificed on day 14.

The primary cause of mortality in mice infected with highly virulent strains was systemic inflammation, particularly neuroinflammation. IL-6 serves as a crucial marker of systemic inflammation induced by highly virulent infections [35]. Serum samples were collected on the third day post-infection prior to mouse sacrifice, and IL-6 levels in serum were measured via an enzyme-linked immunosorbent assay. Our results revealed a significant increase in IL-6 secretion in the mice infected with PRV-Fa, while IL-6 secretion in the mice infected with PRV-ΔTK/ΔgE or PRV-∆TK-(CD2v)-∆gE-(p54) was comparable to the control group (Figure 2d).

**Figure 3:**
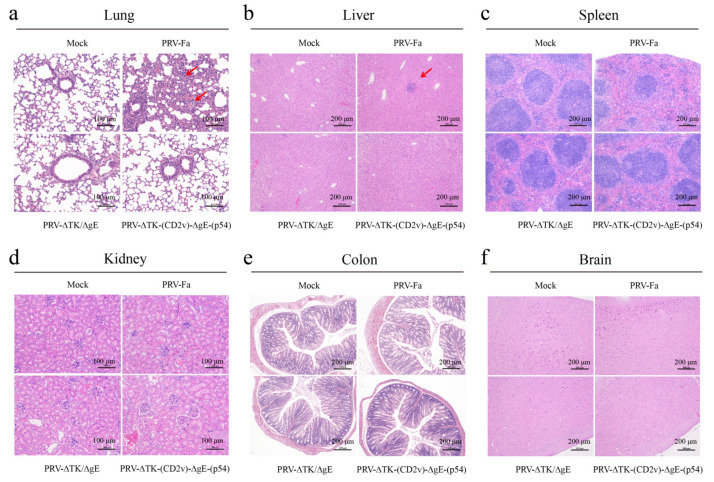
Recombinant PRV protects mice from tissue injury and the reduction in lymphocytes in the white pulp of the spleen caused by PRV infection. To assess the safeguarding potential of the recombinant PRV, mice were infected with wild-type (PRV-Fa), PRV-∆TK-∆gE and PRV-∆TK- (CD2v)-∆gE-(p54) viruses, respectively, by intramuscular injection into the leg (100 μL, 5 × 10^5^ TCID_50_); PBS was used as a control. Mice were sacrificed on day 3 after infection before they died in the PRV-Fa infection group. H&E staining was used for assessment. (**a**,**b**) PRV-Fa infection induced infiltration of immune cells in the lung and liver (red arrow) but not in the other two infection groups, indicating that systemic inflammation was caused by PRV-Fa infection. (**c**) PRV-Fa infection leads to a significant reduction in lymphocytes in the white pulp of the spleen, indicating that PRV-Fa infection depletes lymphocytes in mice. In contrast, the mice infected with PRV-ΔTK/ΔgE or PRV-∆TK-(CD2v)-∆gE-(p54) were unaffected. (**d**–**f**) Kidney, colon, and brain tissues showed no discernible effects. H&E, hematoxylin and eosin; scale bar, 100 µm (lung and kidney) or 200 µm.

In order to evaluate organ injury in the infected mice, we sacrificed mice on the third day post-infection before the mice died in the PRV-Fa infection group and collected the brain, heart, lung, liver, kidney, colon, and spleen. H&E staining revealed that PRV-Fa infection prompted infiltration of immune cells into the lung and liver, a phenomenon not observed in the other two infection groups. This strongly suggests that systemic inflammation is a direct consequence of the PRV-Fa infection. Conversely, PRV-ΔTK/ΔgE or PRV-∆TK-(CD2v)-∆gE-(p54) infection did not induce infiltration of immune cells (Figure 3a,b). Furthermore, PRV-Fa infection leads to a significant reduction in lymphocytes in the white pulp of the spleen, indicating that PRV-Fa infection depletes lymphocytes in mice. In contrast, the mice infected with PRV-ΔTK/ΔgE or PRV-∆TK-(CD2v)-∆gE-(p54) were unaffected (Figure 3c). Nonetheless, the kidney, colon, and brain showed no discernible effects (Figure 3d–f). Moreover, the viral genome DNA was detected in all tissues in PRV-Fa-infected mice but not in the PRV-ΔTK/ΔgE or PRV-∆TK-(CD2v)-∆gE-(p54)-infected groups on the third day (Figure S3). These findings demonstrate that the double knockout of *TK* and *gE* effectively abolishes systemic inflammation triggered by PRV-Fa infection, and an overexpression of CD2v and p54 proteins did not alter the histopathology of PRV-ΔTK/ΔgE.

These results revealed that the recombinant pseudorabies virus PRV-∆TK-(CD2v)-∆gE-(p54) is safe for mice.

### 2.3. Double Knockout of TK and gE Protects Mice from Exhaustion of Multiple Immune Cells Caused by PRV Challenge

In consideration of the role of CD2v in inhibiting lymphocyte proliferation in response to mitogens during ASFV infection [28], we assessed the leukocyte populations in the peripheral blood of immunized mice. Peripheral blood samples, collected on the third day post-infection prior to sacrifice, were processed with Gey’s solution to remove red blood cells and resuspended in PBS supplemented with 2% BSA; then, the cells were stained with a combination of B220-APC, Mac-1-PECy7, and Gr-1-PE for B cells and myeloid cells or stained with a combination of CD3-Alexa Fluor 700, CD4-APC, CD8-BUV805, and CD25-FITC for T cells.

**Figure 4:**
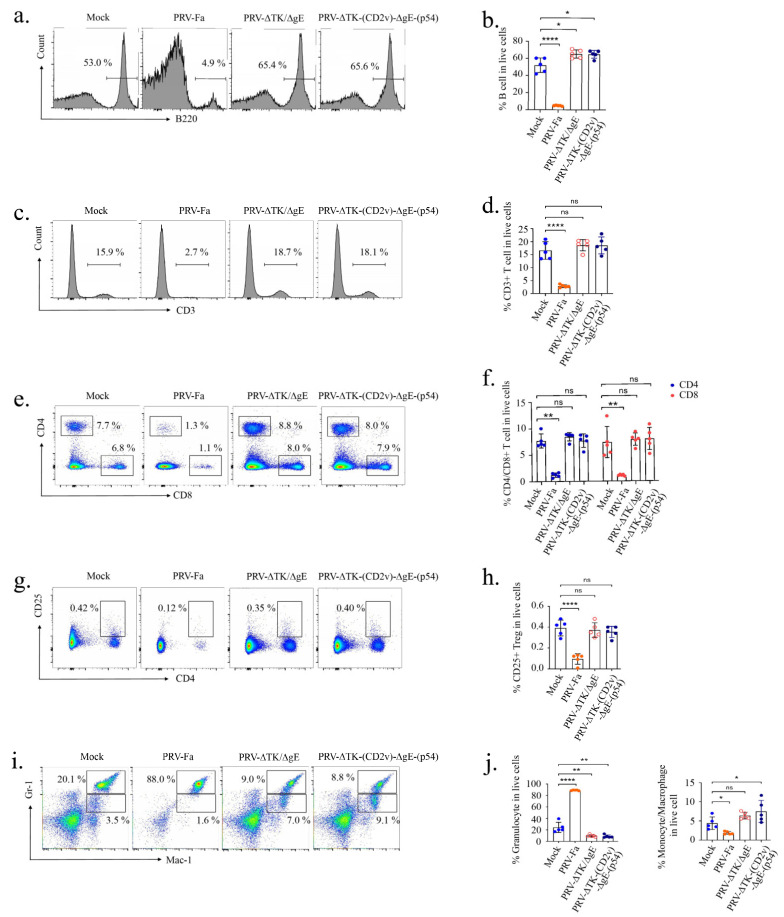
Recombinant PRV protects mice from exhaustion of multiple immune cells caused by PRV infection. (**a**,**b**) The percentage of B cells was markedly reduced in PRV-Fa-infected mice, while PRV-ΔTK/ΔgE or PRV-∆TK-(CD2v)-∆gE-(p54) infections not only prevented this decrease but also stimulated the proliferation of these cells. (**c**–**h**) The percentages of T cells, including CD3^+^, CD4^+^, CD8^+^, and CD4^+^CD25^+^ Treg cells, in PRV-Fa-infected mice were significantly reduced, while the mice infected with PRV-ΔTK/ΔgE or PRV-∆TK-(CD2v)-∆gE-(p54) were unaffected. (**i**,**j**) The percentage of monocytes/macrophages was decreased by PRV-Fa infection but not affected by the infection of PRV-ΔTK/ΔgE, while the infection of PRV-∆TK-(CD2v)-∆gE-(p54) significantly increased the percentage of monocytes/macrophages; in contrast, PRV infection led to an increased percentage of granulocytes, while in the mice infected with PRV-ΔTK/ΔgE or PRV-∆TK-(CD2v)-∆gE-(p54) granulocyte percentage was decreased to a level even lower than the control group. The percentages represent the indicated cells within the gated live cell population. The data presented are derived from five mice in each group. Error bars indicate ± SD. Statistical analysis was performed with the 2-tailed unpaired Student *t*-test. * *p* < 0.05; ** *p* < 0.01; **** *p* < 0.0001; ns, not significant.

The results show that the percentage of B cells was markedly reduced in PRV-Fa-infected mice, while PRV-ΔTK/ΔgE or PRV-∆TK-(CD2v)-∆gE-(p54) infections not only prevented this decrease but also stimulated the proliferation of these cells (Figure 4a,b). Furthermore, our results unveiled a significant reduction in the percentage of T cells, including CD4^+^, CD8^+^, and CD4^+^ CD25^+^ Treg cells, in PRV-Fa-infected mice, while the mice infected with PRV-ΔTK/ΔgE or PRV-∆TK-(CD2v)-∆gE-(p54) were unaffected (Figure 4c–h). Similar to the B cells and T cells, the monocytes/macrophages also experienced a decrease with PRV-Fa infection, but they remained unaffected by PRV-ΔTK/ΔgE infection. Interestingly, PRV-∆TK-(CD2v)-∆gE-(p54) infection significantly elevated the percentage of monocytes/macrophages (Figure 4i,j). In contrast, we observed that PRV infection led to an increased percentage of granulocytes, while the mice infected with PRV-ΔTK/ΔgE or PRV-∆TK-(CD2v)-∆gE-(p54) presented a decrease to a level even lower than that in the control group.

Therefore, wild-type PRV-Fa infection depletes various lymphocytes in peripheral blood. However, the double knockout of *TK* and *gE* in PRV did not impair the immunity of mice. Furthermore, the overexpression of the CD2v and p54 proteins had no discernible impact on the characteristics of PRV-ΔTK/ΔgE.

### 2.4. The Recombinant Pseudorabies Virus-Induced Antibody Production via IM Vaccination in Mice

**Figure 5:**
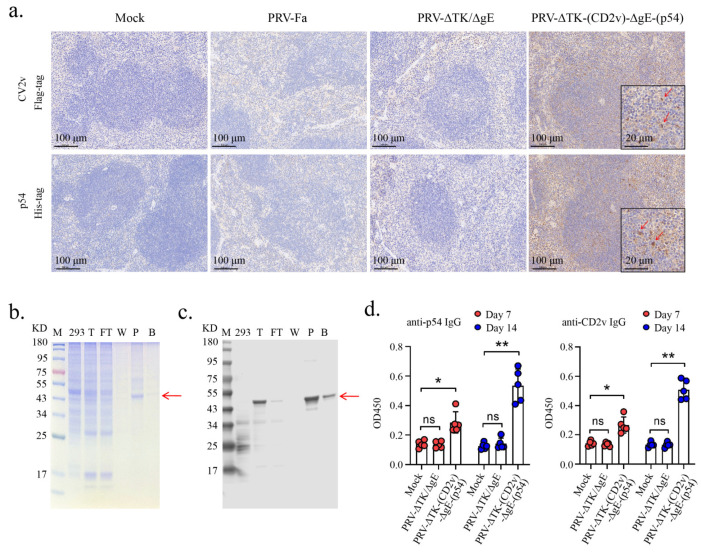
Production of anti-p54 and anti-CD2v-antibodies in mice. (**a**) Mice were infected with PRV-Fa, PRV-∆TK/∆gE, and PRV-∆TK-(CD2v)-∆gE-(p54) by IM injection into the leg (100 μL, 5 × 10^5^ TCID_50_); PBS was used as a control. Post-infection, mice were sacrificed on day 3, and spleens were fixed in 4% paraformaldehyde. Then, paraffin-embedded sections were prepared and subjected to immunohistochemical staining for Flag-tag (**top**) and His-tag (**bottom**), representing the CD2v and p54 proteins, respectively. Scale bar, 100 µm. Insets (scale bar, 20 µm) represent an enlarged local area. The red arrow indicates cells presenting and expressing CD2v or p54 protein. (**b**,**c**) The p54-6×His-tag protein (~45 kD) was purified using a Ni-NTA beads column. Coomassie blue-stained SDS–PAGE (**b**) and Western blot (**c**) of samples from a typical purification of P54 protein from overexpressed 293T cells. Lanes are labeled as follows: M, molecular-weight standards; 293T, untransfected 293T cells; T, total protein; FT, flowthrough (nonbound) from the Ni-NTA beads column; W, protein from the Ni-NTA beads column that was eluted in the wash fractions; P, purified protein after elution from the Ni-NTA beads column; B, Ni-NTA beads after protein elution. The red arrow indicates the target protein. (**d**) PRV-∆TK/∆gE and PRV-∆TK-(CD2v)-∆gE-(p54) were immunized into C57BL/6 mice via IM injection into the leg (100 μL, 5 × 10^5^ TCID_50_). Serum samples were collected on day 7/14 post-immunization for p54- and CD2v-specific IgG detection via ELISA. The results demonstrate that mice immunized with PRV-R produced both p54- and CD2v-specific IgG. The data shown were obtained from five mice in each group. Error bars represent ± SD. Statistical analysis was performed with the 2-tailed unpaired Student *t*-test. * *p* < 0.05; ** *p* < 0.01; ns, not significant.

To ascertain the efficacy of delivering foreign proteins to the spleen via the recombinant PRV, we conducted immunohistochemical staining on spleen sections harvested on the third day post-infection. These sections were treated with anti-His-tag or anti-Flag-tag antibodies. Encouragingly, both CD2v and p54 were distinctly detected in the spleens of mice infected with PRV-∆TK-(CD2v)-∆gE-(p54). Conversely, the mice infected with PRV-Fa or PRV-ΔTK/ΔgE failed to exhibit any detectable expression of either CD2v or p54 (Figure 5a).

To evaluate the immunogenicity of PRV-∆TK-(CD2v)-∆gE-(p54), we successfully expressed and purified the p54 protein from HEK-293T cells (Figure 5b,c). This purified protein served as the substrate for ELISA analysis, enabling the precise detection of p54-specific antibodies. For the CD2v protein, we used the remaining stock from our previous study [32].

Simultaneously, three groups of 6-week-old C57BL/6 male mice underwent IM infection with 5 × 10^5^ TCID_50_ PRV-Fa, PRV-ΔTK/ΔgE, or PRV-∆TK-(CD2v)-∆gE-(p54), while the control group received a PBS vaccination. Antigen-specific IgGs were detected on both days 7 and 14 post-vaccination. The strategy for vaccination is shown in Supplementary Figure S4. Remarkably, PRV-∆TK-(CD2v)-∆gE-(p54) triggered a robust serum IgG response against CD2v and p54 on day 7 after IM vaccination (Figure 5d). Conversely, PRV-Fa and PRV-ΔTK/ΔgE failed to induce serum IgG against CD2v and p54, demonstrating that the recombinant PRV-∆TK-(CD2v)-∆gE-(p54) virus vaccine successfully induced antibody production via IM vaccination in mice. Antigen-specific immunoglobulins (IGs) were also detected and shown in Supplementary Figure S5.

### 2.5. The Recombinant Pseudorabies Virus Confers Effective Protection for Vaccinated Mice after Challenge

Our results demonstrate the immunogenicity and safety of the recombinant PRV-∆TK-(CD2v)-∆gE-(p54) virus vaccine in mice. The next logical step is to evaluate the potential of the vaccine to confer protection against viral infection in mice.

**Figure 6:**
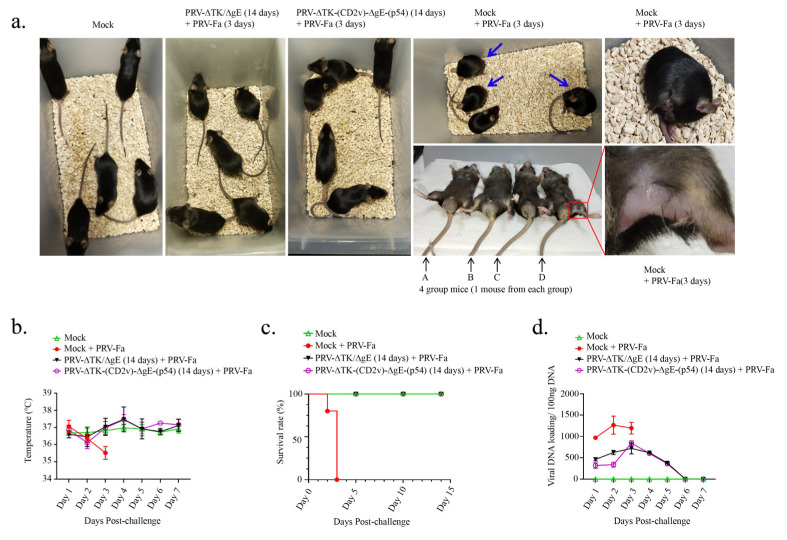
Recombinant PRV provides protection for mice from rapid death caused by the wild-type PRV. (**a**) The behavioral phenotype of mice in each group (*n* = 5) after challenge was observed early from day 3; PRV-Fa caused pruritus in mice vaccinated with PBS and quick death; conversely, mice in the groups vaccinated with PRV-∆TK/∆gE and PRV-∆TK-(CD2v)-∆gE-(p54) were protected from these symptoms. In the group of mice vaccinated with PBS (with a blue arrow), mice curled up and bit the itchy thigh where the virus was injected. The figure at the bottom of the fourth column represents 1 mouse from each group, from left to right: A, mock; B, mouse vaccinated with PRV-∆TK/∆gE and challenged with PRV-Fa; C, mouse vaccinated with PRV-∆TK-(CD2v)-∆gE-(p54) and challenged with PRV-Fa; and D, mouse vaccinated with PBS and challenged with PRV-Fa. The image on the upper right side shows a mouse biting its thigh, and the image on the lower right side shows the depilated thigh of the mouse that bit itself due to pruritus. (**b**) The rectal temperature of mice in each group (*n* = 5) was recorded over the course of 7 days post-challenge; the rectal temperature of the mice in each group was comparable in the first two days, while it decreased in the group of mice vaccinated with PBS on the third day due to the mice being close to death. (**c**) The survival rate of mice in each group (*n* = 5) was recorded for a total of 14 days following the challenge with the corresponding virus; mice were mostly dead by the later part of day 3 in the groups vaccinated with PBS. (**d**) Viral DNA in the feces of mice in each group (*n* = 5, feces of each group mixed together every day) was detected using qPCR after challenge; feces samples were collected for a total of 7 days; viral DNA loading in the PBS-vaccinated group was significantly higher compared to the groups immunized with PRV-ΔTK/ΔgE or PRV-∆TK-∆TK-(CD2v)-∆gE-(p54).

To achieve this, the strategy we used was a homologous prime-boost vaccination regimen. Three distinct groups of mice were subjected to challenge with PRV-Fa via IM injection into the leg (100 μL, 5 × 10^5^ TCID_50_) 14 days after the vaccination period with PBS, PRV-ΔTK/ΔgE, and PRV-∆TK-(CD2v)-∆gE-(p54). The control group was mock-challenged. The strategy for the vaccination and challenge is shown in Supplementary Figure S4. As anticipated, severe pruritus was observed in the PBS-vaccinated group on the third day post-challenge, whereas no such symptoms were noted in the groups vaccinated with PRV-ΔTK/ΔgE or PRV-∆TK-(CD2v)-∆gE-(p54) (Figure 6a). The rectal temperature of mice vaccinated with PRV-ΔTK/ΔgE or PRV-∆TK-(CD2v)-∆gE-(p54) remained consistently stable. In stark contrast, the rectal temperature of mice vaccinated with PBS exhibited a gradual decline and died (Figure 6b). Furthermore, every mouse in the PBS-vaccinated group died on the third day of inoculation. In sharp contrast, mice vaccinated with either PRV-ΔTK/ΔgE or PRV-∆TK-(CD2v)-∆gE-(p54) exhibited robust survival until they were humanely sacrificed on day 14, which was comparable with the control group (Figure 6c).

Meanwhile, we assessed the detoxification effects in mice following the PRV-Fa challenge. Viral genomic DNA in the feces of each group was detected via qPCR for a total of 7 days post-challenge. Notably, viral DNA loading in the PBS-vaccinated group was significantly higher compared to the groups immunized with PRV-ΔTK/ΔgE or PRV-∆TK-∆TK-(CD2v)-∆gE-(p54) (Figure 6d). This suggests that PRV-ΔTK/ΔgE and PRV-∆TK-(CD2v)-∆gE-(p54) do not undergo rapid replication in immunized mice. Therefore, the recombinant PRV confers highly effective protection to vaccinated mice following challenge.

## 3. Discussion

The most efficacious experimental vaccine candidates for the current ASFV strain pandemic are live-attenuated virus vaccines developed by deleting specific virulence-associated genes [10,11,36,37,38]. Despite this, concerns persist regarding their side effects and potential safety issues, including lymphadenopathy, recurrent fever, chronic viremia, persistent chronic infections, and the possibility of virulence recovery [10,11,12,13]. In contrast, the advantages of recombinant virus vaccines are their predictability of response and higher efficacy [14]. Among them, PRV is a good vector for developing multivalent vaccines [15].

Many genes that are unnecessary for PRV replication, such as *TK*, *gE*, and *gI*, can be replaced by foreign genes [25]. The primary virulent genes in PRV, *gE*, and *TK* can be deleted, leading to substantial reductions in invasiveness and virulence while retaining the vaccine’s immunogenicity. Therefore, current commercial live-attenuated PRV vaccines are developed based on the deletion of *gE* and *TK* [9,39,40]. Furthermore, studies indicate that by replacing *gE* and *TK* with foreign genes, the virulent PRV can be transformed into a safe and effective recombinant virus vector vaccine [32,41].

CD2v and p54 play pivotal roles in the infection, internalization, transport, and assembly of ASFV [26,27,33,34]. Consequently, CD2v and p54 represent focal points in the exploration of subunit vaccines, nucleic acid vaccines, and viral vector vaccines.

In this context, we engineered a stable recombinant virus, PRV-∆TK-(CD2v)-∆gE-(p54), using CRISPR/Cas9 technology. This virus expresses both the ASFV CD2v and p54 proteins. Remarkably, PRV-∆TK-(CD2v)-∆gE-(p54) effectively delivers CD2v and p54 and exhibits reduced virulence. Immunization with PRV-∆TK-(CD2v)-∆gE-(p54) neither induces pruritus nor causes systemic infection and inflammation.

Systemic inflammation, particularly neuroinflammation, emerges as the primary cause of mortality in mice following infection with highly virulent PRV strains. As IL-6 stands out as a major marker for inflammation induced by such virulent infections [35], its levels were measured via an enzyme-linked immunosorbent assay. The results reveal a significant increase in IL-6 secretion in mice infected with PRV-Fa, whereas the IL-6 levels in those infected with PRV-ΔTK/ΔgE or PRV-∆TK-(CD2v)-∆gE-(p54) were comparable to the control group.

Moreover, H&E staining showed that PRV-Fa infection induced the infiltration of immune cells into the lung and liver, as well as a significant decrease in lymphocytes in the white pulp of the spleen. In contrast, PRV-ΔTK/ΔgE or PRV-∆TK-(CD2v)-∆gE-(p54) infections did not induce immune cell infiltration or lymphocyte reduction. This indicates that PRV-Fa infection triggers systemic inflammation and lymphocyte depletion in mice. Conversely, double knockout of *TK* and *gE* eliminates the ability of the PRV to induce inflammation and lymphocyte depletion in mice. Furthermore, the overexpression of CD2v and p54 proteins exerted no discernible impact on the characteristics of PRV-ΔTK/ΔgE.

A previous study showed that inflammation promotes T cell overactivation and eventual exhaustion [42]. This strongly implies that systemic inflammation is the underlying cause of lymphocyte depletion resulting from the PRV-Fa infection. This insight suggests that employing anti-inflammatory agents to mitigate inflammation during PRV infection may alleviate lymphocyte exhaustion, thereby fortifying the immunity of afflicted swine and diminishing mortality.

CD2v is a decisive factor for in vitro ASFV infection of peripheral blood lymphocytes to inhibit lymphocyte proliferation in response to mitogens. In vitro, the ASFV deletion of CD2v and the parental virus exhibited indistinguishable growth characteristics on primary porcine macrophage cell cultures; however, mitogen-dependent lymphocyte proliferation of swine PBMCs in vitro was reduced by 90 to 95% following infection with the revertant virus but remained unaltered following infection with the *CD2v* gene deletion mutant [28]. In another study, the attenuated ASFV with a single amino acid substitution in CD2v, Q96R, induced moderate levels of replication and 100% protection against virulent ASFV in pigs [31]. However, it has also been reported that CD2v immunization alone can provide partial protection in the early stages of ASFV infection [30]. In addition, our previous study showed that the recombinant PRV that only carries *CD2v* provides 100% protective ability, induces CD2v-specific humoral and cellular immune responses in mice, and only slightly interferes with the in vivo proliferation of T cells [32]. Based on these studies, one possible explanation is that CD2v requires other components of the ASFV to perform immunosuppressive functions. Individually, CD2v can be used as an effective immunogen to activate the immune system and produce effective protection against ASFV infection. In this study, leucocyte subsets in peripheral blood were detected via flow cytometry. Consistent with a previous study [32], our results show that the percentages of T cells, including CD4^+^, CD8^+^, and CD25^+^ Treg cells, were markedly reduced in PRV-Fa-infected mice, while those infected with PRV-ΔTK/ΔgE or PRV-∆TK-(CD2v)-∆gE-(p54) exhibited no such effects. Notably, our study reveals that PRV-ΔTK/ΔgE or PRV-∆TK-(CD2v)-∆gE-(p54) does not impede T cell proliferation in vivo, suggesting that the double knockout of *TK* and *gE* genes effectively eliminates T cell depletion induced by PRV-Fa infection. Additionally, the overexpression of CD2v and P54 proteins exerts no discernible impact on the characteristics of PRV-ΔTK/ΔgE.

Furthermore, we revealed that PRV-Fa infection can also disrupt B cell proliferation, whereas PRV-ΔTK/ΔgE or PRV-∆TK-(CD2v)-∆gE-(p54) not only refrains from interfering but also stimulates the proliferation of these cells. Prior studies propose that early-stage influenza infection is characterized by substantial upsurges in local type I IFN production, augmenting B cell receptor-mediated proliferation of B cells [43]. Presumably, this aids even low-affinity B cells in orchestrating an antiviral response, thereby initiating the expansion of virus-specific clones [44]. Within two days of type I IFN-induced CD69 expression, infection of B cells within mediastinal lymph nodes induces upregulation of CD69 and the co-stimulatory surface molecule CD86 [45,46], thereby regulating B cell egress from lymph nodes into the bloodstream [47]. The increased B cell levels in mice infected with PRV-ΔTK/ΔgE or PRV-∆TK-(CD2v)-∆gE-(p54) indicate that the double knockout of *TK* and *gE* genes in PRV entirely eliminates the toxicity of PRV-Fa, effectively stimulates the proliferation of low-affinity circulating B cells in peripheral blood, or enhances the ability of B cell egress from lymph nodes into the blood. Moreover, an overexpression of CD2v and P54 proteins does not impact the characteristics of PRV-ΔTK/ΔgE.

Much like B cells, the proliferation of monocytes/macrophages is not only affected by PRV-Fa but also stimulated by PRV-ΔTK/ΔgE or PRV-∆TK-(CD2v)-∆gE-(p54). Monocytes/macrophages constitute a diverse group of cells that serve as the vanguards in innate immunity and, subsequently, as mediators for adaptive immunity to help clear infections [48]. These cells are recognized as professional antigen-presenting cells and “professional” phagocytes, equipped with general receptors and several sensors, particularly pattern recognition receptors that initiate and regulate immune responses against invading pathogens [49]. Increased monocytes/macrophages in mice infected with PRV-ΔTK/ΔgE or PRV-∆TK-(CD2v)-∆gE-(p54) signifies that the double knockout of *TK* and *gE* genes, coupled with recombination of both *CD2v* and *p54* genes in PRV, robustly activate the first responders in innate immunity. In contrast, we revealed that PRV-Fa infection promotes the proliferation of granulocytes while disrupting this in mice infected with PRV-ΔTK/ΔgE or PRV-∆TK-(CD2v)-∆gE-(p54). The percentage of granulocytes decreased to a level that was lower than the PBS control group. According to previous research, increasing immature granulocytes is associated with clinical severity and prognosis after surgery, and in the early phase of sepsis, immature granulocytes could directly contribute to T-cell immunosuppression and thus facilitate secondary post-cardiac surgery infection [50,51]. The observed decrease in granulocytes among mice infected with PRV-ΔTK/ΔgE or PRV-∆TK-(CD2v)-∆gE-(p54) signifies that the double knockout of *TK* and *gE* genes effectively eliminates T-cell immunosuppression from granulocytes, thereby enhancing the ability of the immune system against viral infection. Moreover, the overexpression of CD2v and P54 proteins has shown no discernible impact on the characteristics of PRV-ΔTK/ΔgE.

Moreover, according to another study, CD2v interacts with CSF2RA, which is a hematopoietic receptor superfamily member in myeloid cells and a key receptor protein that activates receptor-associated JAK and STAT proteins. *CD2v* deletion downregulates the JAK2-STAT3 pathway and promotes apoptosis to inhibit ASFV replication [52]. Our study shows that the proliferation rate of monocytes/macrophages in mice infected with PRV-∆TK-(CD2v)-∆gE-(p54) is higher than that in mice infected with PRV-ΔTK/ΔgE, indicating that the recombinant PRV-carrying *CD2v* can specifically promote the proliferation of monocytes/macrophages in myeloid cells rather than granulocytes. The increase in and activation of B cells and monocytes/macrophages, as well as decreased granulocytes, indicates that the recombinant virus can effectively eliminate T cell immunosuppression and activate the immune system in mice, thereby effectively responding to viral infection.

Antibodies play a crucial role in the immune response by fighting off pathogens as well as helping to create strong immunological memory. Pathogen-specific antibodies are a hallmark of an effective immune response following infection or vaccination [53]. To evaluate the immunogenicity of PRV-∆TK-(CD2v)-∆gE-(p54), antigen-specific IGs were detected on days 7 and 14 after vaccination. Remarkably, PRV-∆TK-(CD2v)-∆gE-(p54) elicited significant serum IGs in mice against CD2v and p54 on day 7 following IM vaccination, whereas PRV-Fa and PRV-ΔTK/ΔgE did not elicit serum IGs against CD2v and p54, demonstrating that the recombinant PRV-∆TK-(CD2v)-∆gE-(p54) virus vaccine successfully induced antibody production via IM vaccination in mice. This result shows that PRV-∆TK-(CD2v)-∆gE-(p54) demonstrates good immunogenicity for inducing antibody production against AFSV CD2v and p54, indicating that vaccination with this recombinant virus is able to suppress clinical signs related to PRV infection, while the production of antibodies against CD2v and p54 may offer protection against ASFV infection.

In addition, PRV-∆TK-(CD2v)-∆gE-(p54) confers effective protection for vaccinated mice after challenge. Employing a homologous prime-boost vaccination regimen, severe pruritus and subsequent mortality were observed in the PBS-vaccinated group on the third day post-PRV-Fa challenge. In stark contrast, mice vaccinated with either PRV-ΔTK/ΔgE or PRV-∆TK-(CD2v)-∆gE-(p54) exhibited neither severe pruritus nor mortality until their sacrifice on day 14. This also underscores the potency of PRV-∆TK-(CD2v)-∆gE-(p54) in safeguarding against PRV infection in mice.

## 4. Materials and Methods

### 4.1. Mice

Six-week-old specific-pathogen-free SPF-C57BL/6 male mice were purchased from Wushi Experimental Animal Trade Co., Ltd. (Fuzhou, China) and housed in the Animal Center of Fujian Normal University. All research and animal care procedures were ethically approved by the Animal Ethical and Welfare Committee of Fujian Normal University (IACUC-20230041).

### 4.2. Cells, Viruses, and Plasmids

Vero cells, HEK 293T cells, ST cells, and L929 cells were purchased from the American Type Culture Collection (ATCC). Cell culture reagents were purchased from Life Technologies (Carlsbane, CA, USA), unless otherwise specified. HEK 293T cells, L929 cells, ST cells, and Vero cells were cultured in Dulbecco’s Modified Eagle Medium (DMEM, 11960044, Gibco, New York, NY, USA) supplemented with 10% FBS (Gibco, A5669701), 100 U/mL penicillin, 100 mg/mL streptomycin, and 5% CO_2_ at 37 ℃.

The knockout (KO) plasmids were constructed using the pX459 plasmid vector (Addgene, Boston, MA, USA, 62988). The plasmid was digested with BpiI (37 ℃, 15 min) (Thermo Fisher Scientific, Waltham, MA, USA, ER1011), and sgRNA sequences targeting the PRV gE (5′-GCCGGCGACGATGACCTCAA-3′) and TK (5′-TGCCCGAGCCGATGGCGTAC-3′) genes were designed using the website (http://www.e-crisp.org/E-CRISP/ accessed on 9 October 2021). Following transformation into DH5α *E. coli*, the recombinant plasmids were isolated and verified through sequencing.

The full-length *CD2v* and *p54* genes (based on the pig/HLJ/2018 [1] strain) were synthesized by Wuhan GeneCreate Biological Engineering (Wuhan, China) and used to generate pcDNA3.1(+)-EGFP-Flag-CD2v-Flag and pcDNA3.1(+)-EGFP-6xHis-p54-6xHis. Then, the recombinant vectors were introduced into *E. coli* DH5α (DE3). Plasmids were extracted using a kit purchased from TIANGEN BIOTECH (Beijing, China, DP118-02).

To generate the *CD2v* and *p54* double-knockout virus, PRV-ΔTK/ΔgE transfected the KO plasmids targeting PRV *gE* and *TK* into 5 × 10^5^ HEK 293T cells. Six hours post-transfection, we added 1 × 10^5^ TCID_50_ PRV-Fa. The PRV-Fa strain (GenBank: ON005001.1.: The PRV-Fa strain is the earliest isolated typical strain. It was isolated in the 1960s, and the genomic size was 141,930 nt. This strain caused pseudorabies to become prevalent in China, resulting in most of the cases of pseudorabies and large economic losses in the pig industry [22]), generously provided by the Fujian Academy of Agricultural Sciences, was propagated in Vero cells. The virus culture was harvested once the cytopathic effect reached 90% or more. The TCID_50_ (50% tissue culture infective dose) was determined using a microtiter assay as outlined by Reed and Muench. The sgRNA-induced mutation was verified through PCR and sequencing with specific primers (primers for *gE*-KO are forward 5′-AAAAGGTGGTGTTTGCATAATT-3′ and reverse 5′-TCGGTGGTGATGTAGAACG-3′; primers for *TK*-KO are forward 5′-TCGTAGAAGCGGTTGTGG-3′ and reverse 5′-CGACCAGGACGAACAGG-3′). Four rounds of plaque purification were conducted in the Vero cells to obtain pseudorabies *gE* and the *TK* double-knockout strain PRV-ΔTK/ΔgE.

To generate the recombinant virus PRV-∆TK-(CD2v)-∆gE-(p54), 5 μg of plasmid and 1 μg of the homologous recombinant fragment were co-transfected into 293T cells. Six hours after transfection, we added 5×10^5^ TCID_50_ (100 μL) of PRV-ΔTK/ΔgE. The virus culture was collected when 90% of the cells exhibited cytopathic effects. Finally, the recombinant PRV-∆TK-(CD2v)-∆gE-(p54) strain expressing ASFV CD2v and p54 was amplified and purified in Vero cells through four rounds of phagocytosis. In order to detect the genetic stability of PRV-∆TK-(CD2v)-∆gE-(p54), the virus was passaged to at least 40 generations, and the CD2v and p54 genes were detected by PCR and Sanger sequencing. The recombinant virus was verified through PCR and sequencing with specific primers (primers for *CD2v* are forward 5′-TATTAACACCTGCTACTCCCCCA-3′ and reverse 5′-TTTAGGTAAGGGAAATGGGTTGA-3′; primers for *p54* are forward 5′-GCGGCATTATGGTGAGTG-3′ and reverse 5′-TTATGCGTATAGGTGTTTCTTTG-3′).

To determine the growth kinetics of the virus, 5 × 10^5^ Vero cells in 2 mL of DMEM were seeded into each well of 6-well plates. Then, 1 × 10^3^ TCID_50_ viruses were added to each well, and samples were collected 12, 24, 36, and 48 h post-infection. The virus titer was calculated using the Karber method.

The growth of plaques in Vero cells was observed by crystal violet staining. Vero cells (5 × 10^5^/each well) in the 6-well plates were infected with PRV-Fa, PRV-ΔTK/ΔgE, or PRV-∆TK-(CD2v)-∆gE-(p54) of 2 × 10^2^ TCID_50_ for 36 h, and fixed with 4% paraformaldehyde for 30 min, stained with 2.5% crystal violet. Seven plaques were randomly selected to measure their areas under the microscope. The size of the plaques was determined by ZEN blue 2.3, a picture editing software that comes with the microscope (ZEISS Axio Vert.A1, Carl Zeiss AG, Oberkohen, Germany).

### 4.3. Immunization and Challenge

Six-week-old SPF-C57BL/6 male mice were immunized via intramuscular injection into the leg with PBS, PRV-Fa, PRV-ΔTK/ΔgE, or PRV-∆TK-(CD2v)-∆gE-(p54) (100 μL, 5 × 10^5^ TCID_50_) and then challenged with PRV-Fa (100 μL, 5 × 10^5^ TCID_50_) as a booster 7 days later. The control group received a 100 μL PBS injection.

### 4.4. Viral Copy Analyses in Mice

The viral copy number was assessed in the fecal samples. Fecal matter from the mice was collected every 24 h post-virus challenge. One gram of feces was mixed with 5 mL of phosphate buffered saline (PBS) and soaked for 2 h, and the supernatant was obtained via centrifugation at 3000× *g* for 10 min. This supernatant was used for extracting the genomic DNA for qPCR to determine viral nucleic acid copies. Genomic DNA was extracted and purified using the TIANamp Genomic DNA Kit (TIANGEN BIOTECH, Beijing, China, DP304-03). The qPCR primers were as follows: forward (5′-AACGTCACCTTCGA-GGTGTA-3′) and reverse (5′-AGTCTGAACTCGTGCTTG-3′). The PRV *UL42* gene served as the standard control and was inserted into the pCDH plasmid to create pCDH-*UL42*. The *UL42* gene was also amplified from the genomic DNA of the PRV-Fa strain using the forward primer 5′-ATGTCGCTGTTCGACGAC-3′ and the reverse primer 5′-TTAGAATAAATCTCCGTAGGCG-3′.

The viral copy number was calculated as follows: the average molecular weight (Dalton) of pCDH-*UL42* = base number × 600 (base pair average molecular weight), the length of pCDH-*UL42* = 8542 bp (pCDH) + 1158 bp (PRV-*UL42*) = 9700 bp, and the copy number of 1 ng of pCDH-UL42 = 6.02 × 10^23^ × (1 × 10^–9^/(9700 × 600)) ≈ 1.0 × 10^8^. The standard curve was generated using the copy number of pCDH-*UL42* as the ordinate, and the CT values determined the corresponding CT value as the abscissa, along with the copy number of the PRV genome.

### 4.5. Enzyme-Linked Immunosorbent Assay (ELISA) for IGs and IL-6

To evaluate the resistance to the African swine fever virus, we measured the production of antibodies against p54 and CD2v. PRV-Fa, PRV-ΔTK/ΔgE, and PRV-∆TK-(CD2v)-∆gE-(p54) were administered via intramuscular injections into the leg (100 μL, 5 × 10^5^ TCID_50_) of the C57BL/6 mice. Serum samples were collected on days 7 and 14 post-immunization, and p54- and CD2v-specific antibodies were measured using an enzyme-linked immunosorbent assay (ELISA) (Southern Biotech, Birmingham, AL, USA, 5300-01 and 5300-05).

The safety of the recombinant virus was also evaluated by measuring IL-6 in serum after viral infection. The C57BL/6 mice were infected with PBS, PRV-Fa, PRV-ΔTK/ΔgE, or PRV-∆TK-(CD2v)-∆gE-(p54) via intramuscular injection into the leg (100 μL, 5 × 10^5^ TCID_50_). Serum was collected, and the mice were subsequently sacrificed for experiments on day 3 post-immunization. Serum cytokine IL-6 (FineTest, Boulder, CO, USA, EM0121) levels were measured using an ELISA kit, following the standard protocol.

### 4.6. Hematoxylin–Eosin Staining (H&E Staining) and Immunohistochemistry

Upon the onset of scratching in the control PRV-Fa-infected mouse group (approximately 72 h post-infection), all mice across the groups were humanely euthanized using CO_2_ inhalation. Tissues from the heart, brain, lung, liver, spleen, and kidney were collected. These tissues were fixed with 4% paraformaldehyde for 12 h. Subsequently, the samples underwent a series of ethanol treatments (30%, 50%, 70%, 90%, 95%, and anhydrous ethanol) for 30 min each. This was followed by a transparent treatment involving a mixture of ethanol and xylene (1:1), with xylene ultimately replacing the ethanol in the tissues. The tissues were further treated with a mixture of paraffin and xylene (1:1) overnight before being embedded in the paraffin. Finally, the tissues were sliced into 7 μm sections.

For H&E staining, the sections were first stained with hematoxylin and then with eosin.

Immunohistochemistry was employed to evaluate the protein expression of the recombinant PRV-∆TK-(CD2v)-∆gE-(p54) virus in vaccinated mice. Following the sections of tissues into 7 μm slices, the slices were stained with either mouse anti-Flag-tag (TransGen Biotech, Beijing, Chaina, HT201-01) or anti-His-tag (TransGen Biotech, HT501-01) antibodies. After three washes, the slices were subsequently incubated for 1 h with a 1:5000 dilution of horseradish peroxidase (HRP)-conjugated goat anti-mouse IgG (Beyotime, Shanghai, China, A0216). Following further washes as described above, the slices were stained with 3,3-diaminobenzidine tetrahydrochloride.

The images were captured by scanning with a ZEISS LSM700 microscope (software: ZEN 2.3) (Carl Zeiss AG, Oberkohen, Germany).

### 4.7. Immunofluorescence

Immunofluorescence was used to assess the protein expression of the recombinant PRV-∆TK-(CD2v)-∆gE-(p54) virus in Vero cells. Vero cells (2 × 10^5^) were cultured for 24 h and then infected with the recombinant pseudorabies virus PRV-R (5 × 10^4^ TCID_50_). The cells were harvested 24 h after infection, fixed with 4% paraformaldehyde, and permeabilized with 1 mL of 0.1% Triton X-100 (Sangon Biotech, Shanghai, China, A110694). They were subsequently incubated with mouse anti-Flag monoclonal antibody (TransGen Biotech, HT201-01) or mouse anti-His monoclonal antibody (TransGen Biotech, HT501-01), followed by goat anti-mouse IgG (H+L) cross-adsorbed secondary antibody, Alexa Fluor™ 546 (Invitrogen, Carlsbad, CA, USA, A-11003). The images were captured by scanning with a ZEISS LSM700 microscope.

### 4.8. P54-His Protein Purification and Western Blot Analysis

The pcDNA3.1(+)-EGFP-6×His-*p54*-6×His plasmid was transiently transfected into HEK-293T cells. After 72 h, the p54-6×His-tag protein (~45 kD) was purified using an Ni-NTA beads column. The purity of the recombinant protein was confirmed through Coomassie blue-stained SDS-PAGE and validated using Western blot analysis (probed with mouse anti-His-tag antibody, Proteintech, Wuhan, China, Cat No.: 66005-1-Ig).

Western blotting was further employed to assess the protein expression of the recombinant pseudorabies virus. The Vero, L929, and ST cell lines were infected with the wild-type virus PRV-Fa or PRV-∆TK-(CD2v)-∆gE-(p54) recombinant virus (1 × 10^3^ TCID_50_). After 48 h, the cells were harvested, lysed, subjected to SDS-PAGE, and transferred to PVDF membranes. The membranes were blocked overnight at 4 ℃ with 5% BSA and then incubated with mouse anti-Flag-tag monoclonal antibody (TransGen Biotech, HT201-01) for CD2v detection and mouse anti-His-tag monoclonal antibody (TransGen Biotech, HT501-01) for p54 detection in PBST (PBS supplemented with 0.05% Tween-20 and 5% nonfat milk). Subsequently, the membranes were incubated with goat anti-mouse IgG H&L (Alexa fluor ^®^ 488) (Abcam, ab150117, Cambridge, UK), and the images were obtained by scanning with the Odyssey CLx infrared fluorescence scanning imaging system (LI-COR, Lincoln, NE, USA, 9140-00).

### 4.9. Flow Cytometry

Peripheral blood was collected and treated with Gey’s solution to remove red blood cells. Then, the remaining cells were resuspended in phosphate-buffered saline (PBS) supplemented with 2% fetal bovine serum (FBS). These cells were stained with a combination of fluorescence-conjugated antibodies. Antibodies, including APC-conjugated anti-B220 (553092) and anti-CD4 (553051), PE-Cy7-conjugated anti-Mac-1 (561098), PE-conjugated anti-Gr-1 (561084), BUV805-conjugated anti-CD8 (612898), and FITC-conjugated anti-CD25 (553072), were purchased from BD Biosciences. Alexa Fluor 700-conjugated anti-CD3 (100216) was purchased from Biolegend. The samples were analyzed using the FACSymphony™ A5 cell analyzer (BD Biosciences, Franklin Lake, NJ, USA), and the data were analyzed using FlowJo v10.8 software.

### 4.10. Data Statistical Analysis and Image Processing

The images from the Western blotting, H&E staining, crystal violet staining, and immunofluorescence experiments were processed using Adobe Illustrator CS6 software. All of the experiments were independently repeated at least three times. The data differences between groups were analyzed using unpaired *t*-tests or two-way ANOVA (Graphpad Prism 9.5 Software, San Diego, CA, USA). The data are presented as the mean ± SD for the same treatment.

## 5. Conclusions

In summary, we successfully engineered a recombinant PRV capable of expressing the ASFV CD2v and p54 proteins (PRV-∆TK-(CD2v)-∆gE-(p54)) using CRISPR/Cas9 technology. This recombinant virus exhibits reduced virulence, ensuring safety for mice. It confers effective protection for vaccinated mice after the wild-type PRV-Fa challenge; meanwhile, intramuscular vaccination-induced CD2v- and p54-specific antibodies may offer protection against ASFV infection. Therefore, it holds promise as a potential candidate for the development of a bivalent vaccine. However, further investigations are warranted to confirm its effectiveness in swine populations.

## Data Availability

The data that support the findings of this study are available from the corresponding author upon reasonable request.

## Supplementary Materials

The following supporting information can be downloaded at: https://www.mdpi.com/article/10.3390/ijms25010335/s1.
