# Supplementary material for: Generation and Characterization of Recombinant Pseudorabies Virus Delivering African Swine Fever Virus CD2v and p54
Source: Int J Mol Sci. 2023 Dec 26;25(1):335. doi: 10.3390/ijms25010335 (PMC10779401; doi:10.3390/ijms25010335)
Supplement: Supplementary file 1 [file ijms-25-00335-s001.zip › ijms-2747890-supplementary.pdf]

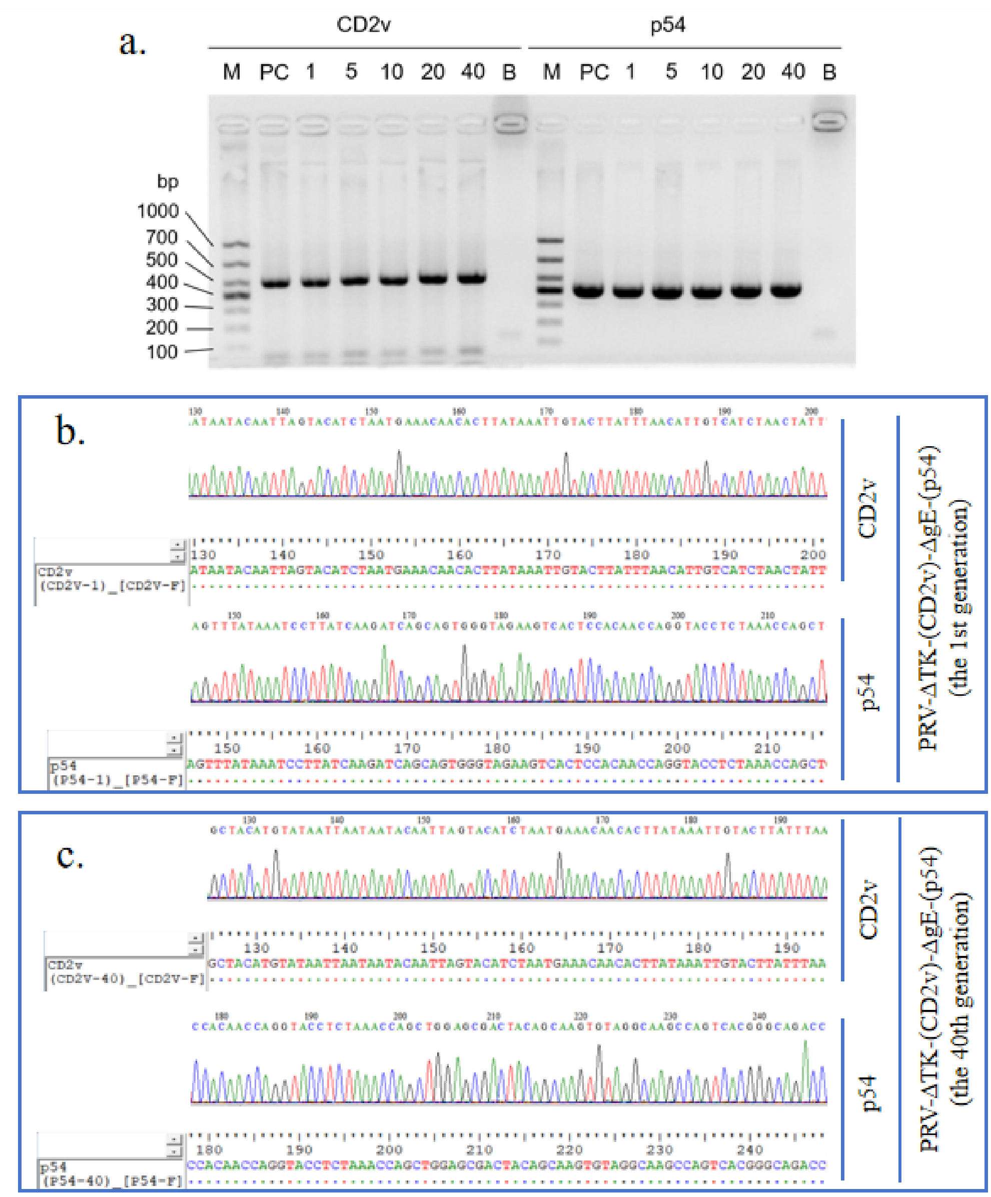


**Supplementary Figure S1:** Genetic stability of PRV-ΔTK-(CD2v)-ΔgE-(p54). In order to detect the genetic stability of PRV-ΔTK-(CD2v)-ΔgE-(p54), the virus were passaged at least 40 generations. (a) Genomic DNA form the $1^{st}$/ $5^{th}$/$10^{th}$/$20^{th}$/$40^{th}$ generation were using as template for genotyping by PCR, *CD2v* (581 bp) and *p54* (501 bp) genes were detected. M, DL1000 DNA marker; PC, positive control by using the recombinant plasmid pcDNA3.1(+)-EGFP-Flag-CD2v-Flag and pcDNA3.1(+)-EGFP-6xHis-p54-6xHis; 1/5/10/20/40, the passage generation of virus; B, blank. (b, c) The PCR products of *CD2v* (upper) and *p54* (bottom) from the first generation (b) and the fortieth generation (c) were using for sanger sequencing. The results showed that the recombinant virus could stably inherit the *CD2v* and *p54* genes.

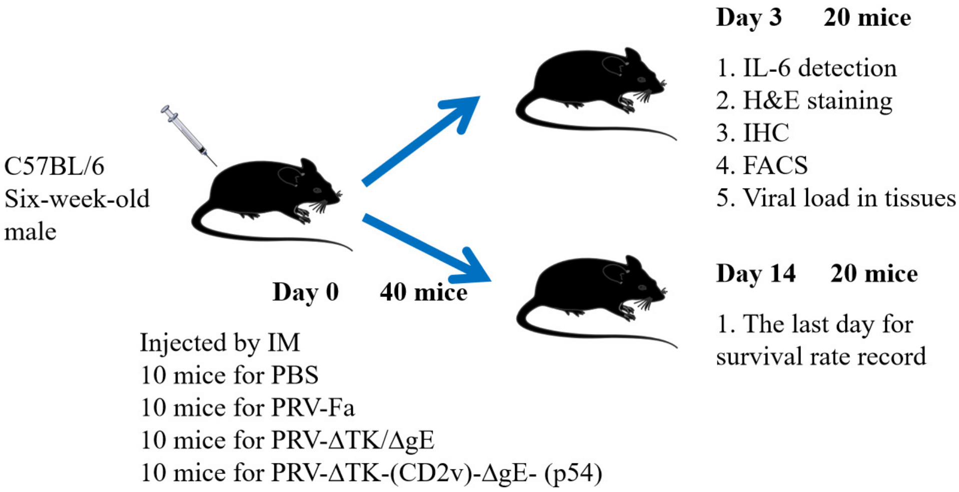


**Supplementary Figure S2:** Strategy for virus infection. Briefly, six-week-old SPF-C57BL/6 male mice were infected via intramuscular injection into the leg with PBS, PRV-Fa, PRV-ΔTK/ΔgE, or PRV-ΔTK-(CD2v)-ΔgE-(p54) (100 μl, $5\times10^5$ $TCID_{50}$) at day 0. (A) 20 Mice were sacrificed at day 3 after infection before they died in the PRV-Fa infection group. (1) Blood sample were collected for FACS analyze; (2) Serum were collected for IL-6 detection; (3 and 4) Lung, spleen, liver, kidney, colon, and brain tissues were collected and fixed in 4% paraformaldehyde, and paraffin-embedded sections were prepared followed by hematoxylin and eosin (H&E) staining or immunohistochemical staining; (5) Genomic DNA from tissuse mentioned above were extracted for viral copy detection. (B) Another 20 Mice were used for suvival reta recording for a total of 14 days.

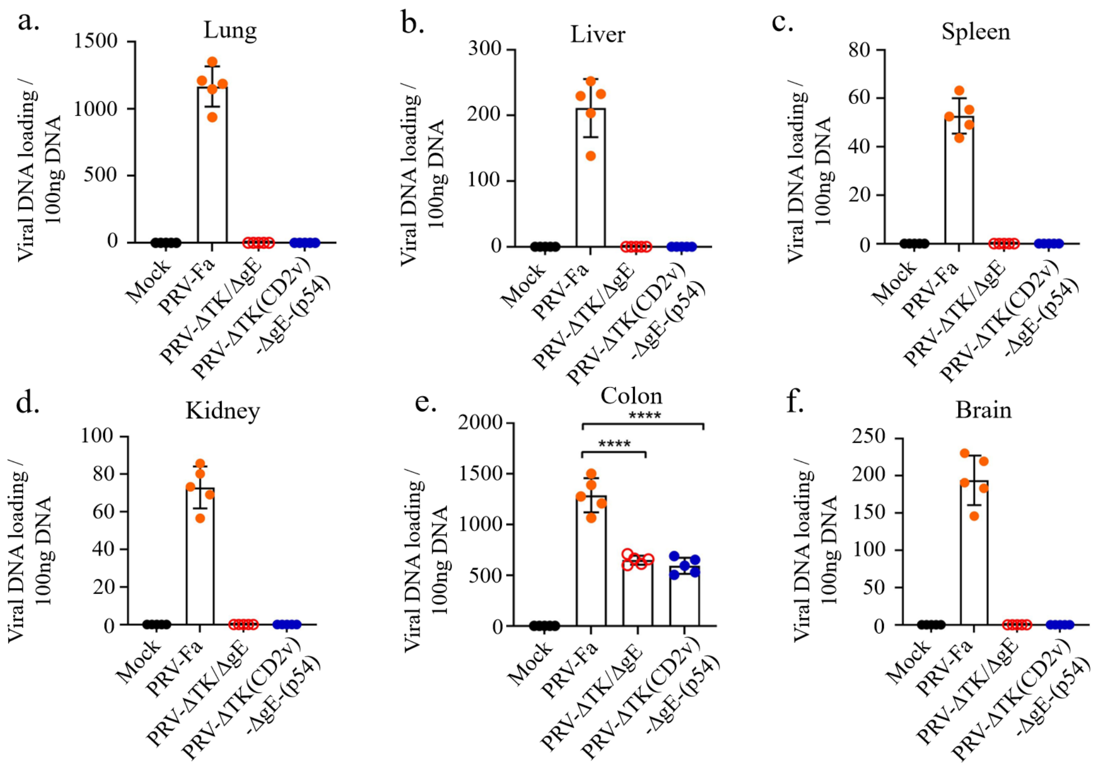


**Supplementary Figure S3:** Viral DNA loading in different tissues. mice were infected via intramuscular injection into the leg with PBS, PRV-Fa, PRV-ΔTK/ΔgE, or PRV-ΔTK-(CD2v)-ΔgE-(p54) (100 µl, $5\times10^5$ $TCID_{50}$) at day 0. Mice were sacrificed at day 3 after infection before they died in the PRV-Fa infection group, Lung, spleen, liver, kidney, colon, and brain tissues were collected and Genomic DNA were extracted, viral DNA loading was detected by qPCR. The virus DNA copies in Lung (a), liver (b), spleen (c), kidney (d), colon (e), and brain (f) were presented.

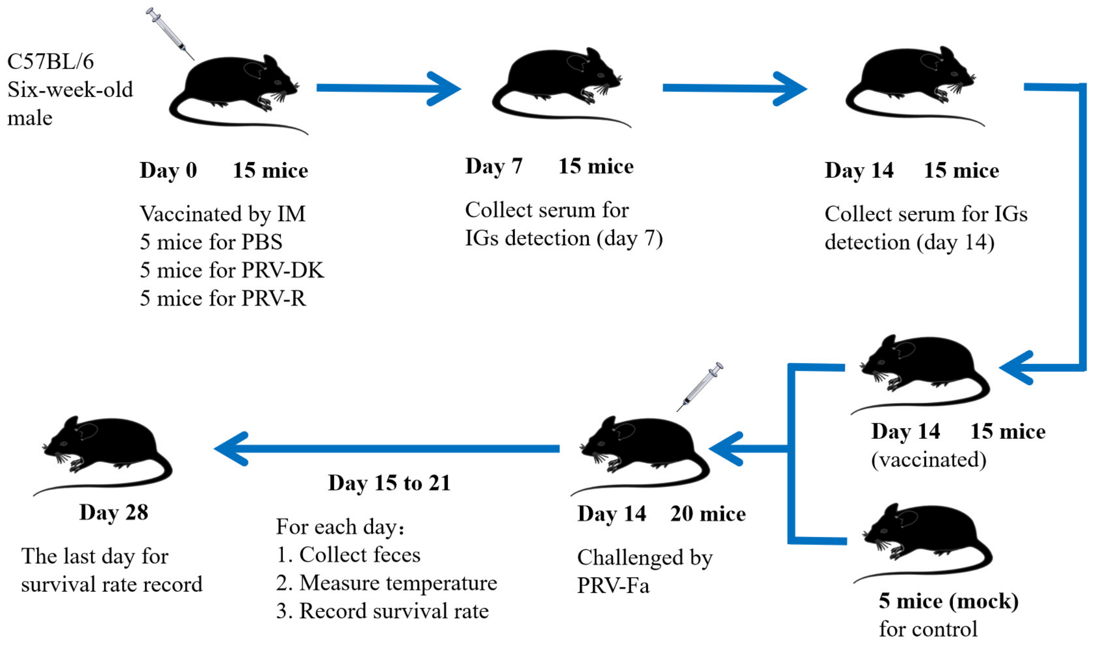


**Supplementary Figure S4:** Strategy for vaccination and challenge. Briefly, six-week-old SPF-C57BL/6 male mice (15 mice, 3 groups) were infected via intramuscular injection into the leg with PBS, PRV-ΔTK/ΔgE, or PRV-ΔTK-(CD2v)-ΔgE-(p54) (100 μl, $5\times10^5$ $TCID_{50}$) at day 0 for vaccination. (A) Serum were collected on day 7 and day 14, CD2v- and p54-specific IGs were detected by ELISA. (B) 15 Mice from (A) were combined with 5 mock mice and challenged with PRV-Fa. (1) feces were collected for viral DNA copy detection; (2) Rectal temperature of mice in each group (n=5) was recorded over the course of 7 days post-challenge; (3) Suvival rate were recorded for a total of 14 days.

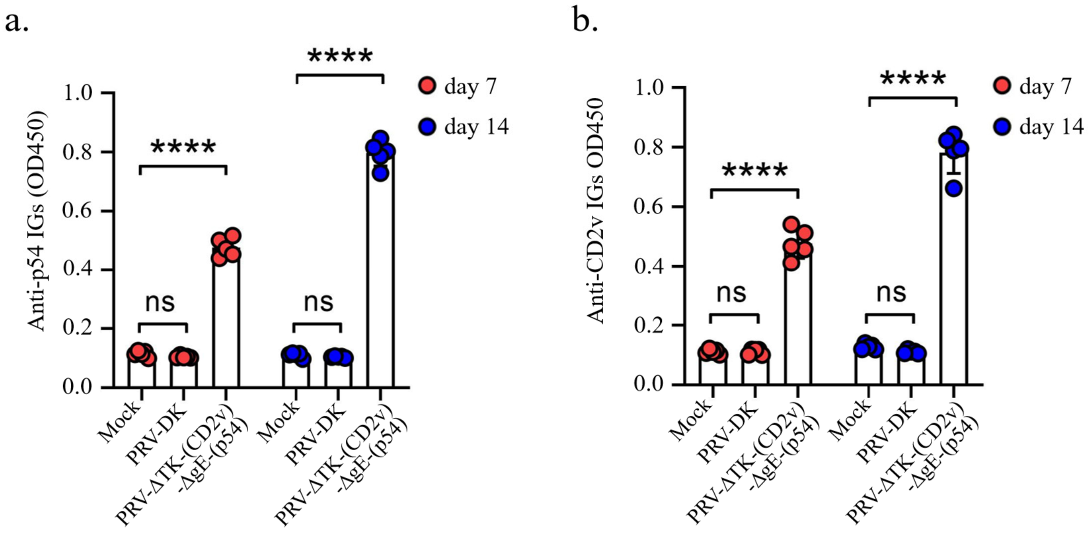


**Supplementary Figure S5:** Production of anti-p54 and anti-CD2v-antibodies. Three groups of 6-week-old C57BL/6 male mice underwent IM infection with $5\times10^5$ $TCID_{50}$ PRV-Fa, PRV-ΔTK/ΔgE, or PRV-ΔTK-(CD2v)-ΔgE-(p54), while the control group received a PBS vaccina- tion. Antigen-specific immunoglobulins (IGs, including IgG, IgA, IgM) were detected both on day 7 and day 14 post-vaccination. p54-specific IGs (a) and CD2v-specific IGs (b) were presented.
